# Immunotherapy for leptomeningeal disease from solid tumors: current clinical outcomes and future opportunities

**DOI:** 10.1007/s10555-024-10235-1

**Published:** 2024-11-29

**Authors:** Eleanor C. Smith, Bryan T Mott, Emily Douglas, Stephen B. Tatter, Kounosuke Watabe

**Affiliations:** 1 Department of Cancer Biology, Wake Forest Baptist Medical Center, Winston-Salem, NC USA; 2 Department of Neurological Surgery, Wake Forest University School of Medicine, Winston-Salem, NC USA; 3 Department of Internal Medicine, Section on Hematology and Oncology, Wake Forest University School of Medicine, Winston-Salem, NC USA

**Keywords:** Leptomeningeal disease, Solid tumors, Immunotherapy, Intrathecal therapy, Immune microenvironment

## Abstract

Leptomeningeal disease is a debilitating, late-stage form of metastatic cancer disseminated within the cerebrospinal fluid, subarachnoid space, and leptomeninges, leading to significant neurological morbidity and mortality. As systemic cancer treatments improve, rates of leptomeningeal disease have increased, yet prognosis remains exceedingly poor. A wide range of treatment modalities have been trialed; however, no standard of care has been established. Additionally, many clinical trials exclude patients with leptomeningeal disease, limiting available prospective data. In this review, we discuss the efficacy of immunotherapy for leptomeningeal disease from solid tumors including systemic and intrathecal therapies, as well as combined therapy regimens. Our review indicates a continued deficiency in the current prospective literature and highlights ongoing research regarding the leptomeningeal immune microenvironment, which will be critical in directing future study of leptomeningeal disease treatment. Currently, the efficacy of immunotherapies on leptomeningeal disease appears limited, and further prospective research is needed to draw significant conclusions. However, recent advancement in understanding the leptomeningeal microenvironment points to potential efficacy of novel immunotherapies targeting the innate immune system, and further study is warranted to evaluate the efficacy of these treatments in this subpopulation of patients.

## Introduction

Leptomeningeal disease (LMD) is an aggressive manifestation of metastatic disease specifically causing dissemination of metastatic cells into the cerebrospinal fluid (CSF), subarachnoid space, and leptomeninges [1–3]. LMD can range from asymptomatic to causing a wide range of neurological symptoms ultimately leading to death [2, 4]. At least 5% of solid tumors progress to LMD, most commonly melanoma, breast, lung, and gastrointestinal cancers, with increasing rates attributed to improved life expectancies with novel systemic therapies [1, 2, 4, 5]. Historically, LMD has been treated by systemic or intrathecal (IT) chemotherapies, with or without whole brain radiotherapy (WBRT), or focused radiotherapy (RT) of bulky lesions [1, 2, 4, 6]. Radiation of the entire neuroaxis is often considered but leads to high risk of neurotoxicity [2]. Despite ongoing improvements in survival of patients with metastatic solid malignancies, survival rates of leptomeningeal disease remain poor, with cohort studies identifying an overall survival of weeks to months. This is likely attributable to the fact that many systemic treatments do not have the ability to penetrate the blood brain barrier (BBB) and target central nervous system (CNS) disease [4, 6–8].

**Fig. 1:**
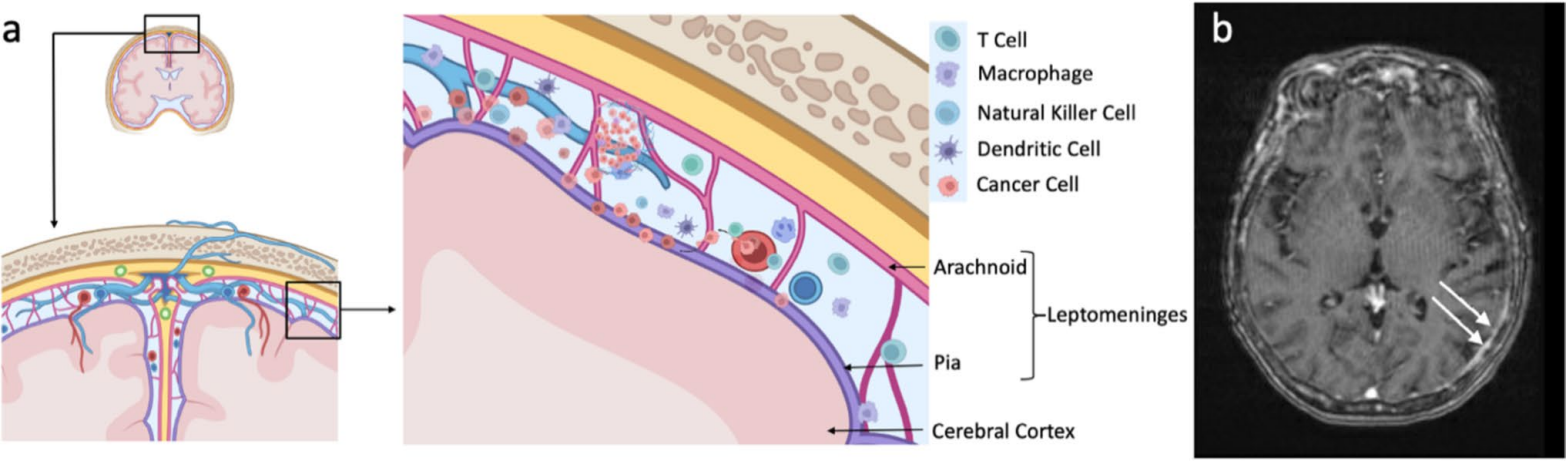
**a** Representation of LMD microenvironment with immune infiltration. **b** MRI of the brain of a patient with LMD from human epidermal growth factor-2 (HER2) positive breast cancer. Dashed arrows indicate leptomeningeal enhancement consistent with LMD (Image created using BioRender.com)

In recent years, understanding of leptomeningeal pathology has advanced. Studies have uncovered a unique and diverse immune landscape within the leptomeningeal space compared to systemic, parenchymal, or even dural immune microenvironments [9] (Fig. 1). Particularly, research has focused on the altered immune response to leptomeningeal tumor infiltration which causes an upregulated inflammatory response. Despite this upregulated immune response, cancer cells continue to thrive, leaving LMD difficult to control [10]. In the non-diseased state, CSF is largely acellular. However, in the setting of LMD, CSF demonstrates myeloid predominant pleocytosis, and the results of single cell sequencing have shown immune-suppressive and tumorigenic M2 polarization of CSF macrophages [10, 11]. Additionally, there is a higher proportion of exhausted T cells and T regulatory cells as compared to control patient CSF without LMD [11].

Many studies have sought to modulate the immune microenvironment of the leptomeningeal space to improve treatment of LMD, particularly with immune checkpoint inhibitors (ICIs) and other novel immunotherapies. In this review, we will discuss the current state of immunotherapy for LMD and review continuing clinical trials of immunotherapy for LMD.

### Early trials of immunotherapy show promise, but conclusions are limited due to small sample size

Long before the development of ICIs, scientists and clinicians sought to harness the immune response against a variety of diseases. Over more than 40 years, knowledge of the immune response, its molecular underpinnings, and mechanisms to harness it have expanded considerably. These strategies have been applied to many oncologic processes including LMD. In a study published in 1987, a group of neurosurgeons in Japan used recombinant interleukin-2 (IL-2) to activate peripheral blood lymphocytes (PBLs) harvested from patients prior to treatment [12]. The resulting “lymphokine-activated killer” (LAK) cells were delivered IT to two patients with LMD. Both patients had improvement in neurologic symptoms and resolution of malignant cells in the CSF following treatment. The first patient lived an additional 7 months, while the second was alive at time of publication 17 months later.

Using tumor-specific antibodies to selectively deliver radioisotopes directly to tumors is another immune therapy approach. In 1990, a study was published applying this technique to 15 patients diagnosed with LMD [13]. Each patient had previously received surgery, chemotherapy, radiation, or some combination thereof. Primary tumors included two patients with melanoma and four patients with carcinoma (ovarian, bladder, breast, and lung) while the remaining patients had primary CNS malignancies or lymphoma. Antibodies that were immunoreactive with the patient’s primary tumor but not with normal CNS cells were radiolabeled with ^131^I and administered IT. Of the two patients with melanoma, progression free survival (PFS) was 8 and 9 months and overall survival (OS) was 12 and 32 months, respectively, with the second patient still alive and without evidence of disease at the time of publication. The patient with bladder carcinoma died 72 h after administration after having multiple presumed seizures, and the patient with ovarian carcinoma was excluded due to protocol deviation. The patient with lung carcinoma had progressive disease with an OS of 4 months, while the patient with breast carcinoma had 26-month PFS and was still alive and without evidence of disease at the time of publication. Common toxicities included aseptic meningitis, bone marrow suppression, and seizures.

Other early immunotherapeutic strategies have employed antibody or cellular therapy. In a study published in 1997, a group from the National Institute of Health (NIH) joined ricin A toxin to the human transferrin receptor and administered this drug IT to eight patients with LMD [14]. The drug was well tolerated, and CSF demonstrated reduction but not complete elimination of tumor cells. Seven of 8 patients had radiographic progression of disease. In 2001, Clemons-Miller et al. published results of a single patient with leptomeningeal melanoma treated with autologous cytotoxic T cells that were stimulated with melanoma-associated antigens *ex vivo*, delivered via right carotid artery injection and subsequently via Ommaya reservoir [15]. The patient had no disease progression during treatment and had improvement of neurological symptoms.

A group from Japan retrospectively evaluated clinical heterogenous treatment of 8 patients with LMD secondary to breast cancer from 1990 to 1999 [16]. All eight patients received WBRT and either no additional treatment, IT methotrexate (MTX), IT adoptive immunotherapy (AIT), or some combination thereof. Three patients received AIT, defined as autologous lymphocytes cultured with tumor supernatant or T cell growth factor. Mean OS of these 3 patients was 6.7 months versus 3.7 months for the 5 patients not receiving AIT. These early studies demonstrate the potential of immunotherapy in LMD and laid the groundwork for more comprehensive studies, though no definite conclusions can be drawn from the small patient numbers and limited controlled evaluation (Table 1).

**Table 1:** Historical Trials of Immunotherapy [12–16]

Title	Author	Year	Study Type	N Total	N Solid Tumor	Pathology	Age	Sex	Treatment	Response	PFS	OS
Adoptive immunotherapy of human meningeal gliomatosis and carcinomatosis with LAK cells and recombinant interleukin-2	Shimizu, K.	1987	Case Series	2	1	SCC	56	M	IT “Lymphocyte Activated Killer Cells”	CR	^17 months	#17 months
Intrathecal administration of 131I radiolabelled monoclonal antibody as a treatment for neoplastic meningitis	Moseley, R. P.	1990	Pilot Study	15	6	2 Melanoma 1 Ovarian 1 Bladder 1 Breast 1 Lung	n/a	n/a	Radioisotope labeled tumor specific antibody	Melanoma CR Ovarian CR Bladder TD Breast CR Lung PD	Melanoma 8, 9 months Ovarian NE Bladder TD Breast ^26 Lung PD	Melanoma 12, #32 months Ovarian NE Bladder 72 hours Breast#26 Lung months
Intraventricular immunotoxin therapy for leptomeningeal neoplasia	Laske, D. W.	1997	Pilot Study	8	7	6 Breast 1 Melanoma	Median 52	n/a	IT immunotoxin 454A12-rRA	Breast 3 PR, 2 PD, 1 NE Melanoma PD	n/a	Breast 26, 10, 3, 3, 3 weeks, 34 h Melanoma 12 weeks
Intrathecal cytotoxic T-cell immunotherapy for metastatic leptomeningeal melanoma	Clemons-Miller, A. R.	2001	Case Report	1	1	Melanoma	49	n/a	Autologous Cytotoxic T Cells	PR	^n/a	#n/a
Meningeal carcinomatosis in patients with breast cancer: report of 8 patients	Yu, H.	2001	Retrospective Review	8	8	Breast	Median 51.5	n/a	4 WBRT alone 1 WBRT+ IT MTX 1 WBRT+ AIT 2 WBRT+ IT MTX+ AIT	WBRT Alone PR, SD, SD, SD WBRT+ IT MTX PR WBRT+ AIT PR WBRT+ IT MTX+ AIT PR, PR	n/a	Total 149.6 days (53–310) WBRT alone 53, 106, 136, 172 WBRT+ IT MTX 110 WBRT+ AIT 213 WBRT+ IT MTX+ AIT 97, 310

*IT* intrathecal, *SCC* squamous cell carcinoma, *CR* complete response, *PR* partial response, *PD* progressive disease, *TD* toxic death, *NE* not evaluated, *MTX* methotrexate, *SD* stable disease

^ No progression at time of publication. # Patient alive at time of publication

### Systemic immune checkpoint inhibitors show mixed effect on LMD, and prospective data is limited

ICIs are a popular form of immunotherapy widely used in solid malignancies that have garnered interest in the treatment of LMD. Immune signals are regulated by the interaction of receptors and ligands on immune cells, antigen presenting cells, and tumor cells. Tumor cells are able to evade the immune system by using these pathways which inhibit antitumor immune response, and antibodies blocking these receptor-ligand interactions help to overcome immune inhibition, improving disease control and survival [17]. Approved therapeutic examples include programmed death-1 (PD-1), cytotoxic T lymphocyte antigen-4 (CTLA-4), and programmed death-1 ligand (PDL-1). Though few prospective studies are currently completed evaluating their efficacy, many retrospective reviews and case reports have attempted to quantify the utility of systemic ICIs in treating patients with LMD. A retrospective review by Hendriks et al. analyzed 19 patients with LMD from non-small cell lung cancer (NSCLC) treated with intravenous (IV) ICIs, including PDL-1 inhibitors with or without anti-CTLA4 antibodies [18]. During ICI treatment, only one patient (5.3%) had improved neurological status with a PFS of 6.4 months and OS of 10.7 months. Nine patients (47.4%) had stable neurological status. Of these patients, 7 (77.8%) had follow up intracranial imaging showing PFS of 1.7–10.4 months, and 2 (22.2%) had progression documented by clinical deterioration at 0.5–0.8 months. OS for these patients ranged from 0.9 to greater than 12.9 months. Nine patients (47.4%) had worsening neurological status during treatment. Of those with intracranial imaging, PFS was 0.7–2.8 months, and the patient without imaging showed a PFS of 0.2 months. OS for these patients ranged from 0.9 to 29.8 months. The patient that lived to 29.8 months died of trauma not related to cancer. These patients were variably on steroids, and those that were receiving this medication the doses varied widely. Nonetheless, their administration did not appear to influence the outcome. Most patients were treated with radiation at some point during their treatment course. The authors concluded that most patients with NSCLC having LMD do not benefit from IV ICI treatment, although a subset obtained longer survival.

A similar review by Zheng et al. describes the course of 32 patients with NSCLC and LMD treated with IV ICIs. A total of six patients received IV ICI combined with other therapies and 26 received monotherapy. After ICI therapy, six patients (18.8%) achieved symptomatic improvement, 14 remained stable (43.8%), and 11 deteriorated (34.4%) [19]. Two patients achieved complete intracranial response, three patients achieved a partial response, and four patients showed stable disease. All other patients showed progressive intracranial disease. Of the two complete intracranial responses, both received single agent ICI. The monotherapy cohort showed median PFS of 2.1 months and median OS of 4.0 months, while the combined cohort showed a median PFS of 3.0 months and median OS of 5.4 months, showing no significant difference between patients undergoing single-agent ICI therapy or combination therapy. Of these patients, 13 were receiving steroids, 19 received prior RT, and one received concurrent RT. No analysis was given on the effect of these additional treatments.

Multiple case reports have additionally evaluated the use of IV ICIs in patients with LMD from NSCLC. Gion et al. report a 54-year-old male with LMD from NSCLC treated with the PD-1 inhibitor nivolumab with improvement in symptoms and clinical PFS of 7 months on ICI, with no discussion of OS [20]. The patient did not receive steroids while on ICI and did not receive RT at all during his treatment. Bover et al. describe a 49-year-old woman diagnosed with LMD from NSCLC achieving complete leptomeningeal response following treatment with IV nivolumab. This patient underwent prior RT but did not receive steroids. She was alive and without progression at 48 months [21]. Further, Arias Ron et al. describe a 48-year-old male with progressive systemic NSCLC and LMD who began IV nivolumab and had improvement in neurological status within one week. He had complete leptomeningeal and systemic response following 6 cycles of nivolumab. At the time of publication, this patient was alive with PFS of 28 months, although nivolumab was discontinued due to inflammatory arthritis [22]. Steroids were given to treat this adverse reaction, but this was late in his therapeutic course. Radiation was initially planned but was held due to clinical response to ICI. A case series by Dudnik et al. evaluated five patients with NCSLC and intracranial disease treated with IV nivolumab, two of which had LMD [23]. These patients had a partial leptomeningeal response and stable LMD with PFS of 7 months and 10 weeks at time of publication, respectively. Importantly, this study specifically excluded patients requiring steroids or that received RT and therefore represents an assessment of ICI response alone. A case series by Vincent et al. reports 3 patients with intracranial metastasis from NSCLC treated with IV PD-1 inhibitor pembrolizumab, one of which had LMD [24]. This patient had complete intracranial response from therapy. She had PFS of 10 months, after which she presented with new brain metastasis, and OS of 13 months from LMD diagnosis. A case by Remon et al. utilized IV atezolizumab, a PDL-1 inhibitor, in combination with bevacizumab and paclitaxel in the treatment of a 73-year-old man with NSCLC and progressive intraparenchymal metastasis and LMD. He had previously undergone RT and was given steroids, but these were held prior to ICI. He had stable disease on imaging and CSF analysis, with improvement in neurological symptoms. This patient had survival of at least 3.5 months from LMD diagnosis at time of publication, though PFS is not reported [25].

Beyond NSCLC, other carcinomas have been treated with ICIs. A case report published in 2020 demonstrated a dramatic response to the addition of IV pembrolizumab in a heavily pre-treated 50-year-old woman with hormone receptor (HR)+ HER2- PD-L1- metastatic breast cancer [26]. She was given two infusions of pembrolizumab with symptomatic improvement within 1 month. Follow up at 8 months demonstrated stable disease and the patient was still alive at publication. An additional case report described a 60-year-old male with diffusely metastatic renal cell carcinoma presenting with worsening back pain [27]. MRI demonstrated spinal metastatic disease including seeding of spinal leptomeninges. He was transitioned to IV nivolumab and demonstrated rapid clinical response with no evidence of LMD at 2 years. Palliative RT was offered but the patient refused this therapy after ICI was started. He did not receive steroids.

The use of ICIs has furthermore gained traction in treating patients with melanoma. A case series by Arasaratnam et al. identifies 14 patients with LMD from melanoma, 8 of which were treated with IV ICIs as monotherapy or in combination with other treatment, 3 who underwent targeted therapy, and 3 who underwent no treatment following LMD diagnosis [28]. Patients treated with ICI monotherapy had OS of 1.5–18.5 months, ICI in combination with other treatment had OS 6.9–12.5 months, targeted therapy had OS 5.7–13.1 months, and no treatment had OS of 0.2–2.8 months. The authors report that those receiving an ICI had increased OS of 7.0 months compared to 5.2 months for the entire cohort. Multiple groups have conducted retrospective reviews of patients in this category and at least one randomized clinical study has been published. A German group retrospectively analyzed 52 patients with LMD from melanoma, 36 of which had concomitant parenchymal lesions [29]. Treatment strategy was very heterogeneous with patients receiving multiple systemic regimens prior to diagnosis with LMD, and only 13 receiving IV ICI alone and 5 receiving IV ICI in combination with targeted therapy. OS in patients receiving systemic therapy of all types was modestly but not significantly increased from 2.9 to 3.7 months and results specifically for those receiving ICI were not provided. A second retrospective study assessed predictors of survival in 51 patients with melanotic LMD, only 12 of which received IV ICI [30]. The data showed a poor response to immunotherapy with the median OS decreasing from 3.6 to 2.9 months, though this was not significant. A third retrospective review from France identified 41 patients with LMD from melanoma, 27 of which received systemic therapy [8]. Of these, 17 received IV ICI alone and 4 received IV ICI plus targeted BRAF inhibitor. This study also evaluated concurrent RT in combination with systemic therapy. Overall, ICI was not associated with significantly improved OS. However, 5 patients receiving both ICI and RT were still alive at least 45 months.

Prospective studies including patients with LMD from melanoma are available. A randomized phase-2 study assessed response to IV ICI monotherapy (nivolumab) or dual therapy (IV nivolumab plus ipilimumab) in treatment naive patients with asymptomatic melanoma brain metastases with a non-randomized arm of patients including 4 with LMD treated with single agent nivolumab [31]. The prognosis was comparatively lower in this cohort and only one patient demonstrated response to therapy.

**Fig. 2:**
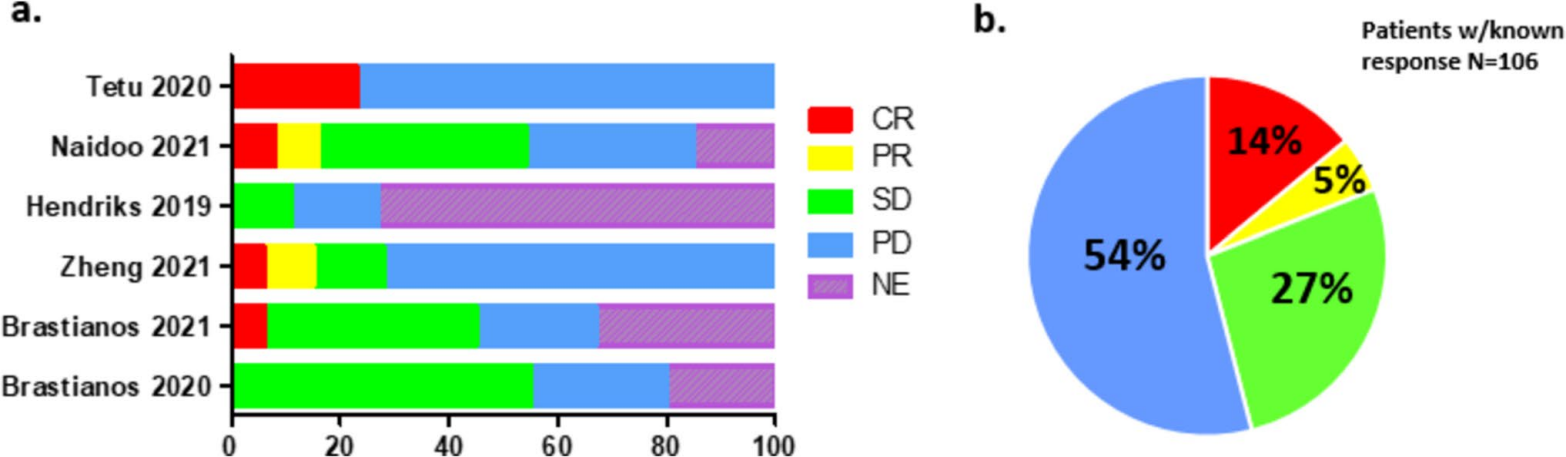
Graphical representation of patient responses to IV ICI treatment regimens. **a** Percent of patients by response to treatment from individual study cohorts. **b** Percent of patients by response to treatment of all cohorts combined. *CR* complete response, *PR* partial response, *SD* stable disease, *PD* progressive disease, *NE* not evaluated (created using GraphPad Prism) [5, 8, 18, 19, 32, 33]

Considerable phase 2 data exists from prospective studies treating patients with LMD without focusing on cancer type. A single-arm phase 2 trial of patients with solid malignancies and LMD were treated with IV pembrolizumab until progression or unacceptable toxicity. Twenty patients (17 with breast cancer, 2 with lung cancer, 1 with ovarian cancer) were included in analysis [32]. All patients had received prior treatment for their cancer, and several remained on additional treatment while concurrently receiving pembrolizumab. The pre-specified primary endpoint was 30% OS at 3 months. Of the 20 patients, 12 (60%) were still alive at 3 months with a median OS for the cohort of 3.6 months. A similar phase 2 study evaluated the combination of IV ipilimumab and nivolumab in the treatment of LMD in 18 patients with solid malignancies [5]. Primary cancer diagnosis was heterogeneous with the most common primary diagnosis of breast cancer (44%) and all patients received some combination of treatment prior to ICI treatment. Eight patients (44%) were alive at 3 months which met pre-specified primary endpoint of 6 patients alive at 3 months (33%). Median OS was 2.9 months. Another study assessed response to IV pembrolizumab in 13 patients having LMD with diverse cancer types. Five patients (38.5%) demonstrated clinical response at 3 months with a median PFS of 2.9 months and median OS of 4.9 months [33]. Based on these studies, response of LMD to IV ICIs appears to be mixed; however, the ability to draw strong conclusions regarding the utility of ICIs is limited due to heterogeneous patient populations and treatments and limited prospective data (Fig. 2).

### Addition of radiation to immune checkpoint inhibitors show promising outcomes, but prospective controlled trials are lacking

Multiple case reports describe the use of ICIs combined with radiation therapies, predominantly WBRT, though to our knowledge, limited controlled trials exist (Table 2). Two reports follow patients with melanoma. One patient, a 63-year-old female, had systemic chemotherapy prior to development of LMD. She then underwent treatment with WBRT and IV ipilimumab and had complete clinical and radiological response, with PFS of 16 months at time of publication and follow up ongoing [34]. The second patient, a 33-year-old male, had undergone prior resection of a brain metastasis, RT, and systemic targeted therapy prior to development of LMD. He then underwent WBRT with stereotactic RT to the spine with subsequent initiation of IV nivolumab. On follow up, he had partial response to treatment with a PFS of 27 months, and OS of 36 months secondary to disease progression [35].

**Table 2:** Immune checkpoint inhibitors combined with radiotherapy [34–38, 40]

Title	Author	Year	Study Type	N Total	N LMD	Pathology	Age	Sex	Treatment	Response	PFS	OS
Clinical and radiological response of leptomeningeal melanoma after whole brain radiotherapy and ipilimumab	Bot, I.	2012	Case Report	1	1	Melanoma	63	F	WBRT+ IV Ipilimumab	CR	^16 months	#16 months
Long-term control of leptomeningeal disease after radiation therapy and nivolumab in a metastatic melanoma patient	Wu, R. C.	2020	Case Report	1	1	Melanoma	33	M	WBRT+ IV Nivolumab	PR	27 months	36 months
Proton craniospinal irradiation with bevacizumab and pembrolizumab for leptomeningeal disease: a case report	Webb, M. J.	2023	Case Report	1	1	Neuroendocrine Carcinoma	60	M	Proton CSI+ Bevacizumab+ Pembrolizumab	PR	4.6 months	7 months
Treating leptomeningeal metastases from primary hepatic neuroendocrine carcinoma with combined radiotherapy and immunotherapy: A case report	Wang, W. J.	2021	Case Report	1	1	Hepatic Neuroendocrine Carcinoma	62	F	WBRT+ IV Nivolumab	CR	^*3 months	3 months
Whole-brain Radiation and Pembrolizumab Treatment for a Non-small-cell Lung Cancer Patient with Meningeal Carcinomatosis Lacking Driver Oncogenes Led to a Long-term Survival	Nakashima, K.	2020	Case Report	1	1	NSCLC	66	F	WBRT+ IV Pembrolizumab	CR	^23 months	#23 months
Targeted treatment and immunotherapy in leptomeningeal metastases from melanoma	Geukes Foppen, M. H.	2016	Retrospective Review	39	39	Melanoma	Mean 52.9	23M 16F	IV BRAF/MEK inhibition IV Ipilimumab RT (SRT or WBRT) Combination No Treatment	1 BRAf/MEK inhibition PR 1 WBRT+ Ipilimumab CR Others NE	n/a	Ipilimumab 6 weeks Ipilimumbab+ RT 47 weeks BRAF/MEK+ RT 25 weeks BRAF 16 weeks RT 4.3 weeks No Treatment 2.9 weeks

*SRT* stereotactic radiotherapy, *WBRT* whole brain radiotherapy, *IV* intravenous, *RT* radiotherapy, *CR* complete response, *CSI* craniospinal irradiation, *NSCLC* non-small cell lung cancer, *NE* not Evaluated

^ No progression at time of publication. # Patient alive at time of publication. * Death from non-neurologic cause

Four other case reports assess WBRT in combination with ICI in patients with NSCLC, primary hepatic neuroendocrine carcinoma, or urothelial carcinoma, respectively. The patient with NSCLC, a 66-year-old female, underwent chemoradiation and second line chemotherapy and stereotactic brain radiation after development of a metastatic brain lesion. Despite this, the patient progressed to LMD. She underwent WBRT and initiation of IV pembrolizumab with improvement in neurological symptoms and imaging findings, and was alive without progression at 23 months at time of publication [36]. A patient with neuroendocrine carcinoma, a 62-year-old female, progressed to LMD after 2 years of treatment. She underwent WBRT with IV nivolumab, with improvement in neurological symptoms and intracranial imaging. Unfortunately, progression of hepatic disease and septic shock ultimately lead to death 3 months from diagnosis of LMD, though she had no LMD progression during this time [37]. A second case report of a 60-year-old male with LMD from neuroendocrine carcinoma treated with ICI and RT has been published. This patient underwent previous resection and radiation of brain metastasis prior to developing LMD, after which he underwent craniospinal irradiation combined with bevacizumab, with addition of IV pembrolizumab to bevacizumab after completion of radiation. He had progression free survival of 4.6 months, but developed hypophysitis induced by pembrolizumab, with rapid neurological decline and ultimate OS of 7 months [38]. IV pembrolizumab and WBRT was used in a patient with urothelial cancer who developed LMD 28 months after starting initial therapy. On this treatment, OS was 4 months [39].

**Fig. 3:**
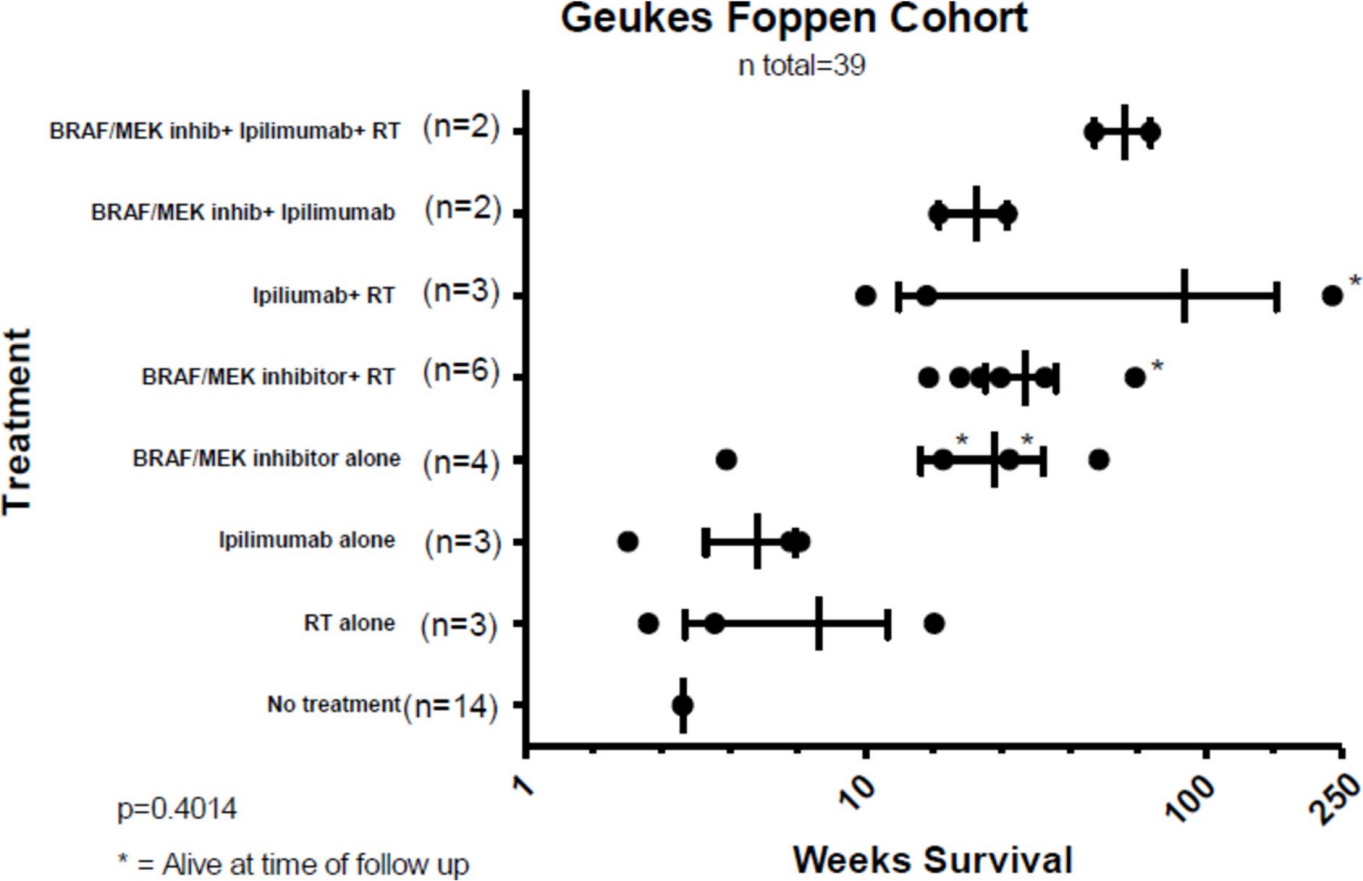
Graphical representation of overall survival of 39 patients in the Geukes Foppen Cohort published in 2016. * Indicates patients who were alive at last follow up. (Created using GraphPad Prism) [40]

Beyond case reports, a review by Geukes Foppen et al. evaluated 39 patients with LMD from melanoma with or without concurrent brain metastases. Of these patients, 25 underwent treatment and 14 completed no treatment following diagnosis. Treatments included IV BRAF/MEK inhibition, IV ipilimumab, RT (whether stereotactic RT or WBRT), or a combination of these. Of the patients treated with IV Ipilimumab, median OS was 47 weeks with RT, and 6 weeks without RT. OS of patients receiving IV BRAF inhibitors was 25 weeks with RT, and 16 weeks without RT. RT alone had an OS of 4.3 weeks. Though BRAF inhibition alone had improved survival following diagnosis of LMD as compared to ICI alone, combination with RT improved the efficacy of both. There was more substantial increase in efficacy when combined with IV ipilimumab, albeit this was not statistically significant [40] (Fig. 3).

Though their low cohort volumes and retrospective nature limit these reports, there may be a survival advantage when combining IV ICIs with RT or WBRT. Treatment benefit must be weighed against the neurotoxicity of RT and WBRT which are estimated to be up to 20% in stereotactic radiosurgery and up to 40% in WBRT [41].

### Treatment with intrathecal immune checkpoint inhibitors is feasible with a favorable safety profile

Though the previous studies outline some clinical improvement in patients with LMD treated with systemic ICIs, the size of these ICIs may limit their effect in the CNS. These monoclonal antibodies are greater than 140,000 Daltons and may have limited ability to cross the BBB, which typically requires a specific mechanism for transport of molecules with a molecular weight greater than 400–500 Daltons [42, 43]. To overcome the limitation of the BBB, IT administration is a mechanism of interest, either by lumbar puncture or Ommaya reservoir placement. In a case series by Huppert et al., two patients with melanoma-derived LMD received IT nivolumab. The first patient, a 49-year-old man developed LMD after treatment with oral targeted chemotherapy and concomitant IV ipilimumab plus nivolumab. Combination IV and IT nivolumab was started, with improvement in radiologic findings, CSF cytology, and clinical status. The second patient, a 39-year-old woman, developed LMD after treatment with IV pembrolizumab and subsequent multiple lines of oral targeted chemotherapy. She initiated IT nivolumab and restarted her primary oral targeted chemotherapy. Following this treatment, she had improvement in her neurological symptoms and tumor cells were not detected in the CSF, though she had continued evidence of LMD on radiographic imaging. Both patients had no significant adverse effect from treatment with IT nivolumab, though it is unclear if their improved clinical picture was from IT ICI treatment, or their simultaneous systemic treatment regimens.

An additional case report by Ensign et al. documents the treatment of a patient with triple negative breast cancer with IT pembrolizumab. Despite multiple systemic treatment modalities, she developed diffuse LMD involving the brain and spine, which progressed on systemic pembrolizumab and high dose systemic methotrexate. She underwent IT pembrolizumab therapy without evidence of adverse treatment events, though unfortunately discontinued treatment due to clinical decline. OS was 6 weeks following initiation of IT pembrolizumab. Lack of post-treatment imaging limits radiographic evaluation of response to IT therapy though it appeared to be well tolerated [44].

Recent results from a phase 1/1b study of concurrent IT and IV nivolumab in 25 patients with LMD from melanoma showed no dose-limiting toxicities and median OS of 4.9 months with 26% of patients still alive at 52 weeks. This trial demonstrated safety of IT ICI in the treatment of LMD and insight on the ideal dosing schedule with emphasis on the importance of well-designed clinical trials in this space [45]. The OS of 4.9 months is comparable to two prior trials of IV pembrolizumab and ipilimumab plus nivolumab, which showed OS of 3.6–4.9 and 2.9 months, respectively [5, 32]. The authors suggest that IT ICI administration may overcome resistance in patients who were refractory of systemic treatment by overcoming the BBB via IT administration. Interestingly, though penetration of the BBB via IV administration is often limited, there are reports of other antibodies (such as trastuzumab) rapidly detected in systemic circulation after intrathecal administration alone [46]. Should this phenomenon hold true for additional antibodies such as nivolumab, it may streamline treatment strategies while also targeting disease outside of the CNS.

Overall, the above studies show promising results in terms of the feasibility and safety of IT ICIs for the treatment of LMD (Table 3). Encouragingly, preliminary results show that IT therapy may overcome resistance to treatment in those with progressing LMD, but further study is warranted.

**Table 3:** Intrathecal immune checkpoint inhibitors [43–45]

Title	Author	Year	Study Type	N Total	N Solid Tumor	Pathology	Age	Sex	Treatment	Response	PFS	OS
Treatment of Metastatic Melanoma With Leptomeningeal Disease Using Intrathecal Immunotherapy	Huppert, L. A.	2020	Case Series	2	2	Melanoma	49 39	1M 1F	1 IV+ IT Nivolumab 1 IT Nivolumab	IV+IT Nivolumab CR IT Nivolumab PR	n/a	n/a
Safety and feasibility of intrathecal pembrolizumab infusion in refractory triple negative breast cancer with leptomeningeal disease	Ensign, S. P. F.	2021	Case Report	1	1	Breast	55	F	IT Pembrolizumab	n/a	n/a	6 weeks
Concurrent intrathecal and intravenous nivolumab in leptomeningeal disease: phase 1 trial interim results	Glitza, I.C.	2023	>Phase 1 Trial	25	22	Melanoma	Median 43	14M 11F	IV+ IT Nivolumab 5mg, 10mg, 20mg, 50mg	n/a	n/a	4.9 months

*IV* intravenous, *IT* intrathecal, *CR* complete response, *PR* partial response, > trial ongoing

### Alternative forms of immunotherapy showed promising response in patients with LMD but adverse side effects limit utility

Multiple studies have investigated alternative forms of immunotherapies for the treatment of LMD (Table 4). Previous research has shown increased infiltration of immune cells into the CSF following administration of IL-2 [47–49]. A review by Glitza et al. analyzed patients with LMD from melanoma treated with IT IL-2 via a compassionate new drug study. The study included 43 patients with a median OS of 7.8 months with 1-year, 2-year, and 5-year OS rates of 36%, 26%, and 13%, respectively. This demonstrates a subset of patients that achieved long-term survival with IT IL2, though administration may be limited due to toxicity [50, 51].

**Table 4:** Alternative forms of immunotherapy [50, 52–54]

Title	Author	Year	Study Type	N Total	N Solid Tumor	Pathology	Age	Sex (M/F)	Treatment	Response	Median PFS	Median OS	Complications
Retrospective review of metastatic melanoma patients with leptomeningeal disease treated with intrathecal interleukin-2	Glitza, I. C.	2018	Retrospective review	43	43	Melanoma	Median 47	32M 15F	IT IL-2 via Ommaya Reservoir	18 CR/PR 1 SD 23 PD 3 n/a	n/a	7.8 months (0.4–90.8)	Elevated ICP requiring intervention in all patients
Immunotherapy with CpG-ODN in neoplastic meningitis: A phase I trial	Ursu, R.	2015	Phase 1 Trial	29	13 17 other	3 Breast 2 SCLC 6 NSCLC 1 Colorectal Adenocarcinoma 1 Melanoma 15 Glioma 1 Ependymoma 1 Melanocytoma	Median 56	12M 17F	Escalating doses of IT CpG-28 via Ommaya Reservoir	n/a	Total 7 weeks (1–81) Breast 5, 4, 6 SCLC 21 NSCLC 4, 1, 5, 2, 21, 19 Colorectal 2 Melanoma 9	Total 15 weeks (3–300) Breast 7, 5, 18 SCLC 23 NSCLC 8, 3, 10, 4, 23, and 28 Colorectal 3 Melanoma 84	16 Erythema 7 Grade 3 Lymphopenia 11 Seizure 2 Arachnoiditis 2 Confusion/fever 2 Ommaya infection 1 SAH
Intrathecal administration of tumor-infiltrating lymphocytes is well tolerated in a patient with leptomeningeal disease from metastatic melanoma: A case report	Glitza, I. C.	2015	Case report	1	1	Melanoma	52	M	IT Tumor Infiltrating Lymphocytes	SD	5 months	5 months	Morbidity from progressive systemic disease
A phase 2 trial of intra-cerebrospinal fluid alpha interferon in the treatment of neoplastic meningitis	Chamberalain, M. C.	2002	Phase 2 Trial	22	12	3 Breast 5 Lung 1 Prostate 1 Colon 2 Melanoma	Median 56	8M 4F	IT α Interferon	Breast PR All others PD	Breast 8, 24, 40	*Total 18 weeks (5–69)	*16 Arachnoiditis *20 Chronic Fatigue

*IT* intrathecal, *CR* complete response, *PR* partial response, *SD* stable disease, *PD* progressive disease, *NSCLC* non-small cell lung cancer, *SCLC* small cell lung cancer, *SAH* subarachnoid hemorrhage, *ICP* intracranial pressure. *No stratification of solid tumor primary vs. others such as hematological malignancy

A case report by Glitza et al. evaluated the use of IT tumor-infiltrating lymphocytes (TIL) for the management of melanotic LMD. TILs are lymphocytes that migrate from the circulation into a tumor, and higher levels of TILs have been found to correlate with improved prognosis. These TILs can be harvested, expanded, and transfused back into a patient to create and anti-tumor immune response [52]. The case report describes a 52-year-old male who developed refractory LMD despite numerous previous treatments. He underwent harvesting of TILs from a lung nodule, and IT TIL administration followed by systemic TIL administration. He had stabilization of LMD on imaging, though he continued to have progression of intraparenchymal lesions and other systemic lesions. This patient ultimately had an OS of 5 months following initiation of IT TIL for LMD treatment [53].

Furthermore, Ursu et al. completed a phase 1 clinical trial evaluating the safety of subcutaneous and IT CpG-28, an oligodeoxynucleotide containing unmethylated cytosine-guanosine motifs that acts as a toll-like receptor (TLR)−9 agonist [54]. TLR-9 is expressed on plasmacytoid dendritic cells (DCs) in the CSF and its agonism activates an immune system response. Though the study was ultimately powered to evaluate safety during dose escalation, secondary endpoints included PFS and OS. Twenty-nine patients were enrolled in the study, 12 with LMD from solid tumors including 3 breast cancer, 6 NSCLC, 2 small cell lung cancer (SCLC), 1 colon cancer, and melanoma. Following treatment, PFS ranged from 1 week in a patient with NSCLS to 84 weeks in a patient with melanoma. Additionally, there was a trend toward higher OS in patients treated with concomitant bevacizumab with IT CpG-28 compared to other concomitant treatment or IT CpG-28 alone, though this was not statistically significant.

A phase II trial by Chamberlain et al. evaluated 22 patients treated with IT α interferon (αIFN), 12 of which had LMD from solid tumors including breast, NSCLC, SCLC, melanoma, colon, and prostate. All breast cancer patients treated had partial response with PFS of 8, 24, and 40 weeks, with progressive disease seen in the remaining solid tumor patients. The authors do note that response to αIFN was significantly higher in patients with hematological malignancy compared to solid tumors [55].

Overall, these forms of immunotherapy appear to be promising, with median OS in all cohorts exceeding historical OS. Unfortunately, both IT IL-2 and IT TIL have significant toxicities, with the authors noting need for CSF diversion due to elevated intracranial pressure in many of these patients [50, 51, 53]. IT Cpg-28 has a risk of seizures and arachnoiditis [54], and over 70% of patients treated with αIFN had chemical arachnoiditis [55]. Due to these toxicities, the use of these IT administrated medications for LMD is realistically limited.

### Ongoing clinical trials further evaluate ICIs as well as novel immunotherapies in patients with LMD

Multiple clinical trials are actively evaluating immunotherapies for treatment of LMD (Table 5). There are two ongoing phase 1 clinical trials evaluating Chimeric Antigen Receptor T-cell (CAR T) evaluation and dendritic cell vaccine (DCV) in individuals with LMD. The Phase 1 trial “HER2-CAR T Cells in Treating Patients With Recurrent Brain or Leptomeningeal Metastases” (NCT03696030) was initiated in 2018. Patients in this trial will undergo treatment with HER2 CAR T cells, with the primary objective of evaluating safety and recommending phase 2 dosing, while secondary objectives include evaluating the tumor and immune microenvironment following treatment, and clinical benefit. Thirty-nine patients with HER2 positive metastatic disease with parenchymal and/or leptomeningeal metastasis have been recruited, and the trial continues to recruit as of this publication [56]. Preclinical support for this trial comes from work by Priceman et al. published in 2017 evaluating the efficacy of IT and intratumoral CAR T cells with different intracellular costimulatory domains targeting HER2 positive breast cancer metastasis to the brain. In *in vitro* studies, they found robust killing of human cancer cell lines with their experimental CAR T as compared to their control, untransduced CAR T cells. They created an *in vivo* model using an orthotopic tumor xenograft breast cancer model in mice, and when treated with their HER2 CAR T cells they observed significant tumor regression. Forty percent of these mice had evidence of leptomeningeal disease when tumors were delivered intraventricularly, which responded to HER2 CAR T treatment, allowing these mice with LMD similar degree of tumor response and survival as mice with parenchymal tumors alone. They also found that the regional delivery of HER2 CAR T provided improved tumor response as compared to systemic delivery, while limiting toxicity that may come with CAR T therapy, such as multiple organ dysfunction secondary to cytokine storm, through their specific engineered costimulatory domain [57, 58].

**Table 5:** Current, ongoing clinical trials of immunotherapy for LMD [55, 58, 64–71]

Study title	NCT number	Trial type	Treatment
HER2-CAR T Cells in Treating Patients With Recurrent Brain or Leptomeningeal Metastases	NCT03696030	Phase I	IT HER2 CAR T Cells
A First in Human Dose Escalation of Dendritic Cell Vaccine (DCV)	NCT05809752	Phase I	IT HER2/3 Primed DCV
Phase II Trial of Pembrolizumab and Lenvatinib for Leptomeningeal Disease	NCT04729348	Phase II	Pembrolizumab + oral Levatinib
Avelumab with Radiotherapy in Patients with Leptomeningeal Disease	NCT03719768	Phase I	IV Avelumab + WBRT
Clinical Observation of ICI Combined With Recombinant Human Endostatin on Leptomeningeal Metastasis of Lung Cancer	NCT05385185	Phase II	IV Camrelizumab or Subcutaneous envafolimab + IV Endostatin
Efficacy and Safety of Durvalumab in Non-Small Cell Lung Cancer With Leptomeningeal Metastasis	NCT04356222	Phase IV	IV Durvalumab + IT Methotrexate
Immune Check Point Inhibitors Intrathecal Injection in Patients with Leptomeningeal Metastasis in NSCLC	NCT06132698	Phase II	IT Tislelizumab + IT Premetrexed
Intrathecal Double Checkpoint Inhibition	NCT05598853	Phase I	IT and IV Ipilimumab + IT and IV Nivolumab
Intrathecal Application of PD1 Antibody in Metastatic Solid Tumors With Leptomeningeal Disease (IT-PD1/NOA 26)	NCT05112549	Phase I	IT Nivolumab
Lymphodepletion Plus Adoptive Cell Transfer With or Without Dendritic Cell Immunization in Patients with Metastatic Melanoma	NCT00338377	Phase II	IT T Cells, IT IL-2

*HER2* human epidermal growth factor 2, *CAR T* chimeric antigen receptor T, *IT* intrathecal, *IV* intravenous, *ICI* immune checkpoint inhibitor

Additionally, the Phase 1 trial “A First in Human Dose Escalation of Dendritic Cell Vaccine (DCV)” (NCT 05809752) was initiated in 2023. Patients with CSF cytology confirmed LMD from HER2-positive or triple-negative breast cancer will undergo treatment with an intraventricular HER2/HER3 primed DCV with the primary objective of finding the maximum tolerated dose and secondary outcomes of OS and PFS. As of this publication, 18 patients have been recruited, and the trial continues to have active recruitment [59]. This trial is based on the preclinical work of Law et al. who created an *in vivo* model of LMD and LMD treatment using an immunocompetent mouse model with HER2-positive or triple-negative breast cancer (TNBC) LMD (BC-LMD) produced by CSF injection of cancer cells. They have created a mouse model of the Ommaya reservoir, which provides access for repeated intra-CSF injections of medications [60, 61]. Using this model they have attempted to harness the innate immune response, creating three dendritic cell vaccines (DCV) targeting HER2 and HER3, independently and in combination. Their results, published in an abstract in September 2022, show that all three groups of BC-LMD mice had a survival advantage as compared to untreated mice. They additionally found that 71% of HER2 positive and 28% of TNBC models had complete resolution of LMD following treatment with HER2/HER3 DCV. They attribute these findings to infiltration of CD4^+^ and CD8^+^ T cells into the CSF space following DCV treatment, as well as upregulation of IFNγ and IL-18 responses, which are inflammatory cytokines shown to play a role in tumor suppression [10, 62–64].

Both Priceman and Law’s studies provide significant preclinical data supporting the evaluation of engineered immune cell constructs targeting tumor cells for the treatment of LMD. Of interest, both studies found that following DCV or CAR T administration, *in vivo* models were protected against development of tumors following secondary tumor inoculation, even without subsequent immunotherapy treatment. Their preclinical results provide the structure for further clinical trials of novel immunotherapy treatments.

Multiple other ongoing clinical trials are evaluating the use of ICIs in combination with other forms of therapy. The “Phase II Trial of Pembrolizumab and Lenvatinib for Leptomeningeal Disease” (NCT04729348) is evaluating pembrolizumab and lenvatinib in patients with LMD from solid tumors. Primary outcome is survival at 6 months, with secondary outcomes of safety, OS, and PFS. Estimated completion of this study occurred in 2022, and results are pending [65]. “Avelumab with Radiotherapy in Patients with Leptomeningeal Disease” (NCT03719768) is an ongoing trial evaluating the use of avelumab with 3000 centiGray WBRT twice weekly in patients with LMD from non-leukemic primary cancers. Primary outcome is safety and dose limiting toxicity, with secondary outcomes of T cell profile in CSF, and OS. This trial has finished recruiting, with estimated completion in 2025 [66]. The phase 2 trial “Clinical Observation of ICI Combined With Recombinant Human Endostatin on Leptomeningeal Metastasis of Lung Cancer” (NCT05385185) is combining the use of ICIs with recombinant endostatin, an anti-angiogenic factor in patients with LMD from lung cancer. The primary outcome is safety and OS with secondary outcome of PFS and disease control rate. This trial continues to recruit, with estimated completion at the end of 2024 [67]. Another trial is evaluating the ICI durvalumab with IT methotrexate therapy for treatment of patients with LMD from NSCLC (NCT04356222). This is a phase 4 trial primarily evaluating OS, neurological PFS, and adverse outcomes, with secondary outcomes of PFS, response rate, and neurological assessment [68]. Another trial evaluating the use of IT ICI combined with additional therapy is “Immune Check Point Inhibitors Intrathecal Injection in Patients with Leptomeningeal Metastasis in NSCLC” (NCT06132698). This trial is a phase 2 clinical trial combining use of tislelizumab and pemetrexed to evaluate primary outcome of objective response rate over 12 months. Secondary outcomes include PFS, OS, control rate, and adverse events. This trial is not yet recruiting [69].

ICIs alone are under evaluation in two current trials. “Intrathecal Double Checkpoint Inhibition” (NCT05598853) is a phase 1 trial assessing the use of combined nivolumab/ipilimumab in patients with LMD from melanoma and NSCLC. Their primary outcome is safety and evaluation of second phase dosing of the medications, with secondary outcomes of survival, progression, and prognostic factors. This trial continues to recruit, with estimated completion in 2025 [70]. IT nivolumab is being evaluated in a Phase 1 clinical trial (NCT05112549) primarily as a dose finding study, with the secondary measure of OS in patients with all forms of all pathologies of LMD. The trial continues to recruit, with estimated completion in in 2025 [71].

“Lymphodepletion with adoptive cell transfer, with or without dendritic cell immunization against metastatic melanoma” is also under clinical investigation. This study is a phase 2 clinical trial with multiple treatment groups of patients with melanoma who will undergo combinations of treatment with chemotherapy, T cell infusion, high dose IL-2, and DCV (NCT00338377). “Cohort D” includes patients with LMD from melanoma who will undergo treatment with IT T cells and IL-2 with primary objective of evaluating safety of this treatment. Secondary objectives will assess CSF and imaging response and overall T cell and cytokine response in CSF. This study has enrolled 1230 patients, with the estimated completion date in 2030 [72]. This study began enrolling in 2006 and it will be interesting to see how the results are interpreted in the setting of rapidly evolving medical care over the course of 24 years. This may result in significant heterogeneity that may limit the utility of the results.

Unfortunately, two other clinical trials were unable to reach completion. One was investigating a unique form of immunotherapy, with intraventricularly delivered bi-specific HER2 antibody armed activated T cells targeting LMD from breast cancer metastasis. They site slow accrual during the COVID-19 pandemic as the reason for discontinuation (NCT03661424) [73]. An additional study sponsored by Y-mAbs Therapeutics was investigating the use of 177Lu-DTPA-omburtamab, a monoclonal antibody targeting B7-H3 with radioactive labelling, in patients with leptomeningeal disease from melanoma, breast, or non-small cell lung cancer (NCT04315246). This study was withdrawn prior to the recruitment of its first participant [74].

## Conclusions and future directions

LMD is a late stage of metastatic disease with continued dismal prognosis despite major advances in cancer therapies over the previous years. This review evaluated the use of immunotherapies in patients with LMD from solid tumors, noting their overall mixed results on patient survival and outcomes, possible limiting toxicities, and paucity of high-quality prospective trial evidence evaluating safety and efficacy.

While ICIs have been the most prominent form of immunotherapy evaluated in LMD, there is concern that the overall immune landscape in LMD may limit their therapeutic benefit. Studies of immune profile in patients with LMD have shown that while there is robust immune cell expression within the CSF of patients with LMD, the immune profile is highly suppressed with predominant expression of exhausted and inactivated CD8 T cells as compared to patients without LMD [75, 76]. Immune cell profiling performed by Im et al. showed that CSF from patients with LMD showed increased numbers of T cells with high CD38 expression as compared to that of patients without LMD, and additionally had varying levels of PD-1 expression [76]. Additional profiling by Smalley et al. showed that all but one patient included in CSF evaluation with LMD had no improvement in T cell activation profile following treatment with ICI, while the one patient who had response to ICI had a unique CSF immune microenvironment compared to other patients with LMD, which showed a profile more similar to normal CSF [75]. CD38 upregulation and high PD-1 expression on T cells has been seen as a mechanism of tumor cell resistance to ICIs [77, 78], and these findings of increased CD38 expression in CSF of LMD patients along with no response of T cell phenotype to ICI treatment lead to the concern that the underlying immune profile of LMD will likely limit the efficacy of ICI therapy.

Additional evaluation of the CSF immune landscape in LMD performed by Smalley et al. showed high expression of M2 macrophages as compared to brain metastasis and primary tumors [75]. These M2 macrophages are anti-inflammatory and play a role in tumor progression by increasing recruitment of T regulatory cells and inhibiting DCs [79]. Immunosuppressive macrophage signaling is also implicated in the development of antiphagocytic signaling within tumor cells, which is regulated by overexpression of CD47, PD-L1, and beta-2-microglobulin, and allows tumors to avoid macrophage dependent phagocytosis [80]. This unique population of macrophages may serve as a target for alternative forms of immunotherapy, which have not previously been evaluated in the setting of LMD. Many clinical trials are currently ongoing and are evaluating the use of monoclonal antibodies against anti-phagocytic signaling in malignancy, including to CD47 and CD24 [81].

Beyond inhibiting signaling cascades that lead to immunosuppressive microenvironments, the use of CAR T, engineered T cells able to target a specific antigen, has shown significant promise of efficacy against malignancies, particularly hematopoietic malignancy. As discussed, preclinical research by Priceman and others shows promise in the fight against LMD and brain metastases [58, 82, 83]. Given the immunosuppressed profile of T cells in LMD, the use of CAR T for LMD treatment is attractive. Unfortunately, clinical use in solid tumors, including brain metastasis, has been less encouraging [84], though ongoing clinical trials may prove successful for LMD. Investigators must be aware of CAR T induced immune effector cell-associated neurotoxicity syndrome, also known as ICANS, which presents with encephalopathy, seizures, and cerebral edema, and can lead to death in extreme circumstances [85]. In individuals with LMD who are already at risk of neurological decline, ICANS could be even more devastating than in the population of individuals receiving CAR T without LMD, and is a risk that needs to be heavily weighed. Ultimately, further clinical trial evaluation is needed to evaluate the safety and efficacy of CAR T in LMD.

The predominance of M2 macrophages leads to the consideration of manipulating the LMD tumor microenvironment macrophage profile to increase tumor phagocytosis and killing. Chimeric antigen receptor macrophages (CARMs) are engineered for antigen specific phagocytosis. CARMs can be customized for activity against specific antigens, including those common in patients with LMD such as HER2 (breast cancer, lung cancer), or mesothelin (lung cancer), allowing targeted therapy against a given pathology, and may have improved survival in the tumor microenvironment as compared to CAR T cells [86, 87]. They have demonstrated antitumor effect against solid tumors *in vitro* and *in vivo*, as well as positive immune activating effects on surrounding tumor cells in the tumor microenvironment, including resting and activated T cells, M2 macrophages, and DCs [88, 89]. Additionally, *in vivo* study of CARMs engineered against brain metastases from lung cancer have shown ability of CARMs to cross the BBB, a significant advantage against intracranial metastasis compared to CAR T [90]. Currently, the first clinical trials evaluating CARMs in the treatment of solid tumors are in process, though these trials do not include patients with LMD [91, 92]. As the safety of CARMs undergoes validation, this promising modality should be quickly adapted in studies targeting LMD from solid tumors.

Further justification for harnessing the innate immune system in the treatment of LMD comes from research performed by Remsik et al. looking at proteomic and transcriptomic analysis of human CSF in patients with and without LMD. Their analysis finds that there is enhancement of IFNγ in LMD CSF. This IFNγ signaling leads to recruitment and proliferation of DCs, with subsequent downstream effect of natural killer (NK) cell proliferation and antitumor activity in an antigen independent manner [10]. These results are partly echoed by the *in vivo* results evaluating DCV targeting HER2 and HER3 for LMD performed by Law et al. discussed above, and results of the ongoing clinical trial supported by this preclinical work will be key in evaluating the efficacy of this treatment for LMD. Employing NK cells is another form of immunotherapy that has yet to be explored in the setting of LMD. In solid tumors, as well as in the murine model created by Remsik et al., NK cell activity has correlated with improved outcomes. Though limited, study of NK immunotherapy has included allogenic and autogenic NK cells, chimeric antigen receptor NK cells, and bispecific killer cell engagers that create a complex with NK cells in order to improve their killing cell function. Several clinical trials focused on this area are ongoing despite developmental challenges [10]. As information regarding NK therapy evolves, there should be significant interest in its use for treatment of LMD.

LMD remains difficult to treat, with different forms of immunotherapy showing marginal results. As the cellular microenvironment of LMD is further elucidated, novel immunotherapies harnessing the unique immune microenvironment of LMD warrant further investigation. Therapies focused on cells of the innate immune system such as macrophages, DCs, and NK cells show promise based on current knowledge of the LMD CSF microenvironment. We anticipate that these innate immune system therapies will produce the most promising outcomes in the future, while therapies such as ICIs may not show clinically significant effect due to their overall interaction with the LMD immune microenvironment. Ultimately, further development and validation of these novel treatment modalities is necessary. As more patients are presenting with LMD, dedicated clinical trials that evaluate both current and emerging therapies are urgently needed.

## Data Availability

No datasets were generated or analysed during the current study.
